# Draft genome sequence of *Acetobacter persici* DS1, a strain for high-acidity vinegar fermentation

**DOI:** 10.1128/mra.01119-24

**Published:** 2025-01-15

**Authors:** Neha Mani Tripathi, Puneet Kumar Singh, Asha Yadav, Krishna Kant Sharma, Amit Kumar Jain, Gajender Kumar Aseri, Deepansh Sharma, Deepti Singh

**Affiliations:** 1 Amity Institute of Microbial Technology, Amity University Rajasthan 211579 , Jaipur, India; 2 Biomedical Sciences, Chonnam National University Hospital, Chonnam, South Korea; 3 Department of Microbiology, Maharshi Dayanand University, Rohtak, Haryana, India; 4 IGNOU Regional Centre, Karnal, Haryana, India; 5 Department of Life Sciences, J.C. Bose University of Science and Technology, YMCA, Faridabad, India; Rochester Institute of Technology, Rochester, New York, USA

**Keywords:** *Acetobacter persici *DS1, whole genome sequence, sapodilla fruit, vinegar fermentation

## Abstract

The draft genome sequence of *Acetobacter persici* DS1 was isolated for high-acidity vinegar fermentation. The *A. persici* DS1 draft genome has a length of 8,793,442 bp, with 44.27% GC content, 3,053 proteins, 3,169 genes, and 15 genes that play an important role in acetic acid production.

## ANNOUNCEMENT

In the present study, *Acetobacter persici* DS1 was isolated from the Sapodilla (*Manilkara zapota*) fruit on the glucose yeast extract calcium carbonate medium with a pH of 5.5 and incubated at 30°C for 48 h. The conserved region of the 16S rRNA sequence was amplified and deposited in the NCBI GenBank with accession number ON724153. The strain displayed ethanol resistance up to 5.5% (vol/vol) initial ethanol concentrations and 6.5% (vol/vol) for initial acetic acid resistance. Furthermore, the strain displayed the highest level of alcohol dehydrogenase (ADH) and aldehyde dehydrogenase (ALDH) enzyme activity at a concentration of 10% acetic acid. The ADH activity of *A. persici* DS1 was 2.93 U mg^−1^, and its ALDH activity was 3.52 U mg^−1^ (1). Membrane-bound ADH, which depends on pyrroloquinolinequinone, transforms ethanol into acetaldehyde. The membrane-bound enzyme ALDH then rapidly converts the generated acetaldehyde into acetate (2). The strain’s maximum acetic acid production was 4.63 g/100 mL when using absolute ethanol and 3.82 g/100 mL when using distilled ethanol. The genomic DNA was then extracted with Qiagen DNA extraction kit. The whole genome of *A. presici* DS1 was sequenced using Illuminanovaseq 6000. The sequenced reads were quality checked using FastQC version 0.11.9. The *de novo* assembly was completed using Megahit version 1.2.9. The contiguity of the assembled result is increased when long reads are used, resulting in 8,793,442 bp, with 44.27% GC content (*N*_50_; 186,430 bp) and a 96.7% genome coverage, which were assembled into scaffolds, consisting of 1,081 bp minimum contigs and 3,191,180 bp maximum contig length. The NCBI genome of *A. persici* (accession No. GCF_002006565.1) was used as a reference genome, obtained from NCBI Refseq Genome Database that showed 99.93% mapping identity(Table 1). Plastflow, a neural network-based classifier trained on plasmid sequences from the Refseq database, was used to predict the plasmid sequences in the genome. The assembled genome had a size of 202,734 bp and a GC content of 53.44%. The assembly uploaded to NCBI was annotated via PGAP with the annotation method “GeneMarks-2+.” Gene prediction revealed 3,053 proteins, 3,169 total genes, 3 rRNAs, 46 tRNAs, and 4 ncRNAs. Furthermore, five types of ADH genes (EC 1.1.1.1, iron-containing, zinc-containing, zinc type ADH-like protein, and quinohemo protein ADH, Type III) and three aldehyde dehydrogenase genes (EC 1.2.1.3, A EC 1.2.1.22, and EC 1.2.1.3 in 4-hydroxyproline) were identified and are responsible for the production of acetic acid. Additionally, the citrate synthase gene (citrate synthase EC 2.3.3.1), involved in acetic acid resistance mechanism, acetyl-CoA hydrolase (aarc) genes (EC 3.1.2.1 and acetyl-CoA hydrolase transferase family protein), and various terminal oxidase genes (terminal oxidase biogenesis protein CtaM), the genes responsible for acetic acid fermentation, were also found. Furthermore, the cytochrome oxidase gene present in the ethanol respiratory chain (cytochrome oxidase biogenesis protein and heme A synthase, cytochrome oxidase biogenesis) and ATP synthase β gene (ATP synthase β chain EC 3.6.3.14) accountable for ATP generation in acetic acid fermentation process were identified. The reported draft genome sequence of *A. persici* DS1 has potential for vinegar fermentation; therefore, it is ideal for use as a strain suitable for a starter culture for the production of fruit vinegar.

**TABLE 1:** Summary of whole-genome sequence statistics of bacteria *A. persici* DS1

Description	Values
Assembled genome size (bp)	8,793,442
GC content (%)	47.27
Assembly *N*_50_ (bp)	186,430
Genome coverage (%)	96.7
Minimum contig length (bp)	205
Maximum contig length (bp)	427,171
Gap information (%)	0.19
Protein	3,053
Genes	3,169
Acetic acid production genes	15
Total number of predicted coding sequences	3,532

Earlier, various *Acetobacter* whole genomes were reported for the production of acetic acid (3–5). The GenDB software was used for gene discovery and annotation. *Acetobacter pasteurianus* 386B genome yielded 2,595 and 280 protein-coding sequences for the chromosome and plasmids, respectively (Table-1) (6). PlasFlow has a 96% accuracy rate in recovering plasmid sequences from assembled metagenomes without requiring any prior knowledge of the samples’ functional or taxonomic makeup. Additionally, it can perform preliminary taxonomical classification of sequences and recover sequences from both circular and linear plasmids. Genes that contribute to the genetic diversity of bacterial populations, such as those governing characteristics like acid tolerance, can be carried by plasmids (7). According to the NCBI Prokaryotic Genome Annotation Pipeline, *Acetobacter tropicalis* has 3,383 genes that encode for 3,215 proteins (8).

## Data Availability

This draft genome research has been cited at DDBJ/ENA/GenBank with accession number JBHIKZ000000000. The draft genome data for Acetobacter persici DS1 are available through NCBI under BioProject ID PRJNA1161488 with BioSample accession number SAMN43782066 and Sequence Read Archive accession number PRJNA1161488.
